# The role of dermatoscopy and reflectance confocal microscopy in the assessment of relapsing secondary cutaneous follicular B-cell lymphoma

**DOI:** 10.1016/j.jdcr.2022.03.035

**Published:** 2022-04-12

**Authors:** Alessandro Laghi, Claudia Lee, Alexander Witkowski, Maylee Hsu, Giovanni Pellacani, Joanna Ludzik

**Affiliations:** a Dermatology Clinic, Department of Clinical Internal, Anesthesiological and Cardiovascular Sciences, Sapienza University of Rome; b Dermatology Unit, Department of Medicine, “Celio” Military Hospital, Rome, Italy; c Department of Dermatology, Oregon Health and Sciences University, Portland, Oregon; d School of Medicine, University of California Riverside, Riverside, California; e Department of Pathology, Veterans Affairs Portland Health Care System, Oregon Health & Science University, United States Veterans Hospital, Portland, Oregon; f Department of Dermatology, University of Modena and Reggio Emilia, Modena, Italy; g Department of Telemedicine and Bioinformatics, Jagiellonian University Medical College, Krakow, Poland

**Keywords:** dermatoscopy, follicular lymphoma, reflectance confocal microscopy, skin cancer, tumor relapse, PCFCL, primary cutaneous follicular center-cell lymphoma, RCM, reflectance confocal microscopy, SCFL, secondary cutaneous follicular lymphoma

## Clinical presentation

**Fig 1:**
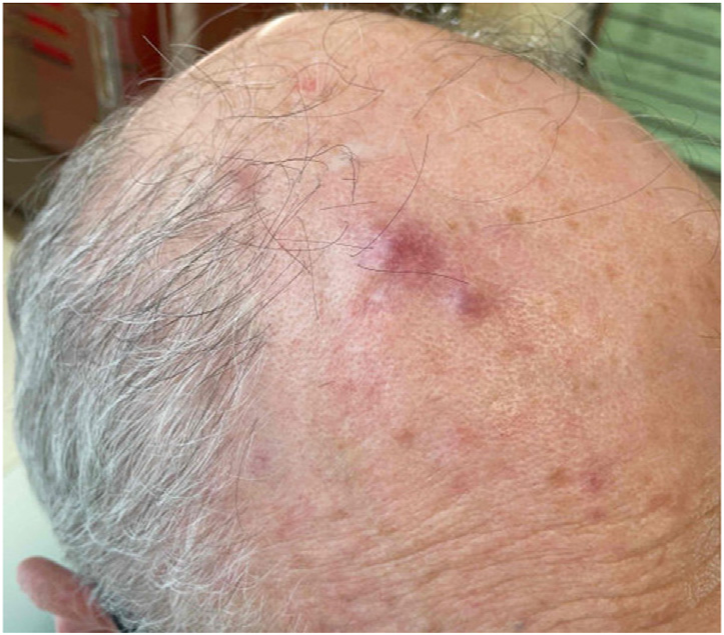
Clinical picture demonstrating 5-15–mm erythematous-to-purple patches and nodules on the scalp.

A 72-year-old man presented with several infiltrated painless patches and nodules (5-15 mm) on his scalp, without lymphadenopathy. The patient reported that he previously had similar erythematous-to-purple lesions and had been treated with a 2-week course of topical steroid, reporting a partial response (Fig 1).

Nine years previously, findings from a histopathologic examination performed on a retroperitoneal lymph node were consistent with a low-grade B-cell follicular lymphoma. The patient’s original bone-marrow biopsy was inadequate; however, flow cytometry identified a monoclonal B-cell population. Due to the absence of symptoms and the low tumor burden, the oncologist decided not to follow up with the patient and did not prescribe any therapy. Before being seen in our clinic for the first time, the patient developed scalp lesions that were previously confirmed to be histologically consistent with an atypical B-cell lymphoid population that regressed spontaneously.

During his visit with our team, we performed dermatoscopy and reflectance confocal microscopy (RCM; Vivascope 1500; Caliber Imaging and Diagnostic, Inc) of the scalp lesions, which, in consideration of the clinical history, corroborated the diagnosis of relapsing secondary cutaneous follicular lymphoma (SCFL). SCFL is a systemic B-cell non-Hodgkin lymphoma that involves the skin, with a variable clinical course. The neoplastic cells are derived from germinal-center B cells that coexpress CD10, CD19, and CD22, but usually are negative for CD5. Frequently, SCFL presents a t(14;18) (q32;q21) translocation that juxtaposes the BCL2 and immunoglobulin heavy chain genes, leading to overexpression of the antiapoptotic protein, BCL2.^1^ In contrast, primary cutaneous follicular center-cell lymphoma (PCFCL) tends to be negative for BCL2, CD10 and the t(14:18).^2^ PCFCL and SCFL generally share virtually identical cutaneous clinical presentations.^2^

### Dermatoscopic appearance

**Fig 2:**
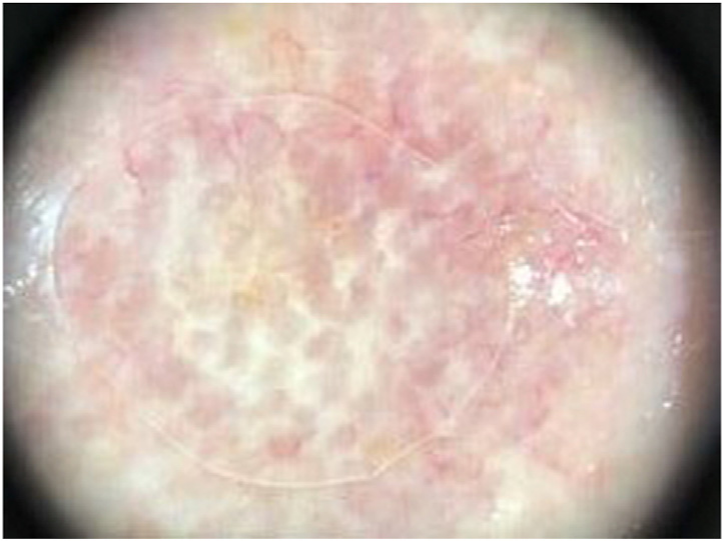
Dermatoscopic features, showing a white pseudonetwork centrally located in the nodules with surrounding telangiectatic vessels on an erythematous background.

To the best our knowledge, no dermatoscopic features have been described about relapsing SCFL or SCFL to date. Our examination disclosed a glittering white pseudonetwork with scales, yellow circles, polymorphic vessels, surrounded by some telangiectatic vessels on an erythematous background (Fig 2, Video 1, available on www.jaadcasereports.org).

These findings are partially shared with PCFCL,^3^ which is not surprising given the extensive histologic similarity.^2^

### RCM findings

**Fig 3:**
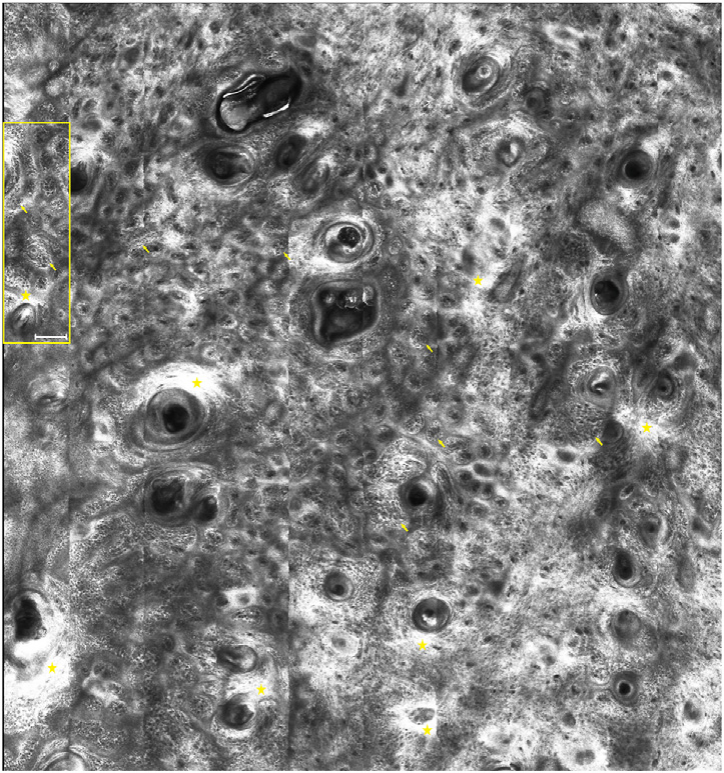
Reflectance confocal microscopy features revealing nucleated cellular infiltration (*yellow arrows*), including appendages with surrounding fibrosis (*yellow stars*). The yellow square represents Fig 4, *C*. Scale bar: 0.25 mm.

RCM has been used for the study of other cutaneous lymphomas, even relapsing ones, but not for PCFCL or SCFL.^4^ In the papillary dermis (107 μm depth recorded on RCM), we observed nucleated cellular infiltration, including appendages with surrounding fibrosis (Fig 3). These findings, while not diagnostic, are interesting, when the nucleated cells are distinguishable from atypical melanocytes. In the absence of atypical melanocytes and melanocytic nests, this information can be used to exclude those types of malignancies and corroborated the diagnosis of cutaneous lymphoma.^2^^,^^5^

### Histologic diagnosis

**Fig 4:**
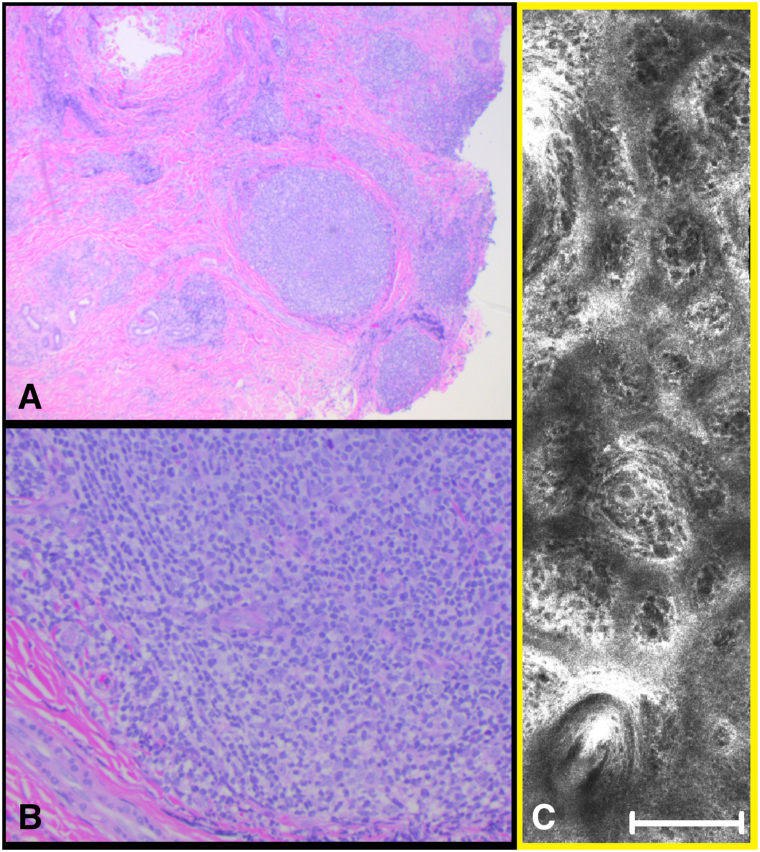
Histologic feature (**A** and **B**, hematoxylin-eosin stain; original magnifications: **A**, ×10; **B**, ×40). **C,** comparison with reflectance confocal microscopy findings.

Sections demonstrated skin with multiple dense lymphoid nodules within the papillary and reticular dermis, with minimal cellular atypia (Fig 4, *A* and *B*). Immunohistochemistry revealed a lymphoid population of CD20^−^ and PAX5^+^ B cells in nodules surrounded by CD3^+^ T cells. B cells were positive for CD10, BCL2, BCL6, and negative for CD5 and cyclin D1. Fluorescence *in situ* hybridization demonstrated IGH/BCL2 fusion.

The histologic findings reassembled the RCM findings (Fig 4, *C*).

### Key message

The clinical presentation of this case postulates several differential diagnoses, including multifocal basal cell carcinoma, amelanotic melanoma with satellites, Merkel cell carcinoma, and Kaposi sarcoma; however, with the additional visualization provided by dermatoscopy and RCM that demonstrated the absence of pathognomonic features, we were confidently able to rule out these suspicions. Given the patient’s history and histologic confirmation, we made the diagnosis of SCLF relapse.

## Conflicts of interest

None disclosed.
